# Serum adiponectin and transient elastography as non-invasive markers for postoperative biliary atresia

**DOI:** 10.1186/1471-230X-11-16

**Published:** 2011-02-28

**Authors:** Sittisak Honsawek, Maneerat Chayanupatkul, Voranush Chongsrisawat, Apiradee Theamboonlers, Kesmanee Praianantathavorn, Wanvisa Udomsinprasert, Paisarn Vejchapipat, Yong Poovorawan

**Affiliations:** 1 Department of Biochemistry, Faculty of Medicine, Chulalongkorn University, Bangkok 10330, Thailand; 2 Department of Physiology, Faculty of Medicine, Chulalongkorn University, Bangkok 10330, Thailand; 3 Center of Excellence in Clinical Virology, Department of Pediatrics, Faculty of Medicine, Chulalongkorn University, Bangkok 10330, Thailand; 4 Department of Surgery, Faculty of Medicine, Chulalongkorn University, Bangkok 10330, Thailand

## Abstract

**Background:**

Biliary atresia (BA) is a progressive inflammatory disorder of the extrahepatic bile ducts leading to the obliteration of bile flow. The purpose of this study was to determine serum adiponectin in BA patients and to investigate the relationship of adiponectin with clinical parameters and liver stiffness scores.

**Methods:**

Sixty BA patients post Kasai operation and 20 controls were enrolled. The mean age of BA patients and controls was 9.6 ± 0.7 and 10.1 ± 0.7 years, respectively. BA patients were classified into two groups according to their serum total bilirubin (TB) levels (non-jaundice, TB < 2 mg/dl vs. jaundice, TB ≥ 2 mg/dl) and liver stiffness (insignificant fibrosis, liver stiffness < 7 kPa vs. significant fibrosis, liver stiffness ≥ 7 kPa). Serum adiponectin levels were analyzed by enzyme-linked immunosorbent assay. Liver stiffness scores were examined by transient elastography (FibroScan).

**Results:**

BA patients had markedly higher serum adiponectin levels (15.5 ± 1.1 vs. 11.1 ± 1.1 μg/ml, *P *= 0.03) and liver stiffness than controls (30.1 ± 3.0 vs. 5.1 ± 0.5 kPa, *P *< 0.001). Serum adiponectin levels were significantly elevated in BA patients with jaundice compared with those without jaundice (24.4 ± 1.4 vs. 11.0 ± 0.7 μg/ml, *P *< 0.001). In addition, BA patients with significant liver fibrosis had remarkably greater serum adiponectin than insignificant fibrosis counterparts (17.7 ± 1.2 vs. 9.4 ± 1.1 μg/ml, *P *< 0.001). Subsequent analysis revealed that serum adiponectin was positively correlated with total bilirubin, hyaluronic acid, and liver stiffness (*r *= 0.58, *r *= 0.46, and *r *= 0.60, *P *< 0.001, respectively).

**Conclusions:**

Serum adiponectin and liver stiffness values were higher in BA patients compared with normal participants. The elevated serum adiponectin levels also positively correlated with the degree of hepatic dysfunction and liver fibrosis. Accordingly, serum adiponectin and transient elastography could serve as the useful non-invasive biomarkers for monitoring the severity and progression in postoperative BA.

## Background

Biliary atresia (BA) is a progressive, inflammatory, fibrosclerotic cholangiopathy resulting in complete obliteration of the extrahepatic bile ducts [1]. The obstruction of bile flow leads to worsening cholestasis, hepatic fibrosis, biliary cirrhosis, end-stage liver disease, and death within a few years [2]. Currently, Kasai operation or hepatoportoenterostomy constitutes the initial surgical treatment of choice for infants with BA. Although Kasai procedure can successfully establish bile flow to the gastrointestinal tract, a number of BA children progress to hepatic cirrhosis, portal hypertension and ultimately require liver transplantation [3]. To date, the etiology and pathogenesis of BA have not been completely understood; however, several mechanisms have been proposed including genetic defects, perinatal viral infections, morphogenic abnormalities, immune mediated bile duct injuries, and autoimmune disorders involving the bile ducts [4,5].

Bile duct inflammation, cytokine responses, and bile acid toxicity are three major contributors of liver parenchymal destruction and hepatic fibrosis in BA patients [2]. After hepatic stellate cells (HSC) are activated, these key effecter cells in hepatic fibrogenesis are transformed into extracellular matrix-producing myofibroblast. This process results in the production and the accumulation of collagen and other extracellular matrix in liver parenchyma, thus initiating and perpetuating the liver fibrosis [6,7]. Recent studies showed the role of adipokines in hepatic fibrogenesis of various chronic liver diseases [8]. In this study, we focused on a unique adipokine, adiponectin.

Adiponectin, a 244 amino acid polypeptide, is the most abundant adipokine exclusively produced and secreted by adipocytes into systemic circulation in trimeric, hexameric, and larger multimeric high-molecular-weight (HMW) forms [9,10]. Adiponectin is structurally homologue to tumor necrosis factor-α (TNF-α); however, these two molecules antagonize each other's effects in the target organs [11]. Adiponectin exerts its anti-inflammatory effects through the reduction of pro-inflammatory cytokines release including TNF-α and interleukin-6, and inducing the expression of anti-inflammatory cytokines, such as interleukin-10 [12,13]. Adiponectin is also renowned for its anti-diabetic, anti-atherosclerotic, and anti-obesity effects. It is believed that adiponectin plays a protective role in liver diseases. In animal studies, adiponectin-knockout mice developed more severe carbon tetrachloride-induced liver fibrosis compared with wild type mice, and adiponectin injection prior to carbon tetrachloride treatment could prevent it [14]. In non-alcoholic obese mice, administration of recombinant adiponectin could attenuate hepatomegaly, hepatic steatosis, and aminotransferase abnormality [15]. Moreover, elevated adiponectin levels correlate positively with the severity of liver cirrhosis and negatively with hepatic protein synthesis [11,16]. In contrast, low adiponectin levels have been shown in non-alcoholic fatty liver disease [17]. Adiponectin levels correlate negatively with liver fat and hepatic insulin resistance in diabetic patients [18]. Adiponectin is currently a subject of research interest since it has the potential to be a useful marker for liver fibrosis, and a possible target for a new therapeutic approach.

Recently, it has been reported that a number of cytokines and growth factors have been studied in BA patients including osteopontin [19], basic fibroblast growth factor [20], and stem cell factor [21]. To the best of our knowledge, there have been no published studied on serum adiponectin levels from various clinical stages of BA. This is the first study to evaluate the correlation of serum adiponectin, liver stiffness and clinical outcomes in postoperative BA. In the present study, we postulated that serum adiponectin could be associated with the severity of clinical outcomes and the liver stiffness in BA patients, and to prove this hypothesis, we analyzed serum adiponectin and liver stiffness in BA patients compared with healthy controls. Therefore, the purpose of this study was to determine serum adiponectin levels collected from BA patients and to examine the possible correlations of serum adiponectin and outcome parameters of postoperative BA patients.

## Methods

All parents of children were informed of the purpose of the study and of any interventions involved in this study. Written informed consents were obtained from participants' parents prior to the children entering the study. This study complied with the ethical guidelines of the 1975 Declaration of Helsinki, and was approved by the Institutional Review Board of the Faculty of Medicine, Chulalongkorn University.

### Study Population

Sixty BA patients (32 girls and 28 boys with mean age of 9.6 ± 0.7 years) and 20 healthy children (10 girls and 10 boys with mean age of 10.1 ± 0.7 years) were recruited in this study. All BA patients had undergone hepatic portojejunostomy with Roux-en-Y reconstruction (original Kasai procedure), and they were generally in good health; no signs of suspected infection or bleeding abnormalities at the time of blood sampling. None of them had undergone liver transplantation. Healthy controls who attended the Well Baby Clinic at King Chulalongkorn Memorial hospital for vaccination had normal physical findings and no underlying disease. BA patients were classified into two groups according to serum total bilirubin (TB), serum alanine aminotransferase (ALT), and liver stiffness score. Based on their jaundice status, BA children were divided into a non-jaundice group (TB < 2 mg/dl) and a persistent jaundice group (TB ≥ 2 mg/dl). Subsequently, BA patients were categorized into a non-significant fibrosis group (liver stiffness < 7 kPa) and a significant fibrosis group (liver stiffness≥7 kPa). The cut-off point of liver stiffness score for significant fibrosis was based on the study by Castera L, et al. [22] with sensitivity of 67% and specificity of 89%.

### Laboratory methods

After overnight fast, samples of peripheral venous blood were collected in the morning from every participant, centrifuged for 15 min at 1000 × g, and stored immediately at -80°C for further analysis. Quantitative determination of adiponectin concentration in serum was performed using commercially available enzyme-linked immunosorbent assay (ELISA) (R&D Systems, Inc., Minneapolis, MN, USA). According to the manufacturer's protocol, 50 μl of recombinant human adiponectin standards and serum samples were pipetted into each well, which has been pre-coated with specific antibody for adiponectin. After incubating for 2 h at room temperature, every well was washed thoroughly with wash buffer for 4 times. 200 μl of a horseradish peroxidase-conjugated monoclonal antibody specific for adiponectin was then added to each well and incubated for a further 2 h at room temperature. After 4 washes, substrate solution was pipetted into the wells and then microplate was incubated for 30 min at room temperature with protection from light. Lastly, the reaction was stopped by the stop solution and the color intensity was measured with an automated microplate reader at 450 nm. The adiponectin concentration was determined by a standard optical density-concentration curve. Twofold serial dilutions of recombinant human adiponectin with a concentration of 3.9-250 ng/ml were used as standards. The manufacturer reported precision was 2.5-4.7% (intra-assay) and 5.8-6.9% (inter-assay). The sensitivity of this assay was 0.246 ng/ml.

The liver function tests including serum albumin, total bilirubin (TB), direct bilirubin (DB), aspatate aminotransferase (AST), alanine aminotransferase (ALT), and alkaline phosphatase (ALP) were measured using a Hitachi 912 automated machine at the central laboratory of our hospital. The aspartate aminotransferase to platelets ratio index (APRI) was calculated as follows: (AST/upper limit of normal) × 100/platelet count (10^9^/l) [23].

### Liver stiffness measurement

Transient elastography (FibroScan) measured the liver stiffness between 25 to 65 mm from the skin surface, which is approximately equivalent to the volume of a cylinder of 1 cm wide and 4 cm long. The measurements were performed by placing a transducer probe of FibroScan on the intercostal space at the area of right lobe of liver with patients lying in a dorsal decubitus position with maximal abduction of right arm. The target location for measurement was a liver portion that was at least 6 cm thick, and was free of major vascular structures. The measurements were performed until 10 validated results were achieved with a success rate of at least 80%. The median value of 10 validated scores was considered the elastic modulus of the liver, and it was expressed in kilopascals (kPa).

### Statistical analysis

Statistical analysis was performed using SPSS software version 16.0 for Windows. Mann-Whitney U test was used to compare the difference of serum adiponectin concentrations between groups. Correlation between serum adiponectin levels and other serological markers, and liver stiffness scores were calculated using Pearson's correlation coefficient (*r*). Data were expressed as mean ± SEM. *P*-values < 0.05 were considered to be statistically significant.

## Results

### Comparisons between BA patients and healthy controls

**Figure 1:**
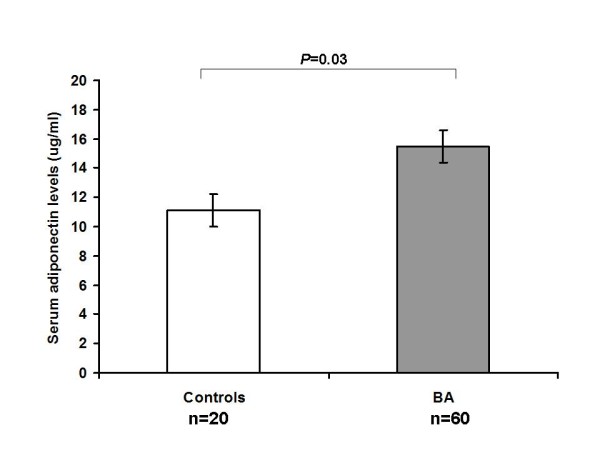
**Comparison of serum adiponectin levels in biliary atresia patients and healthy controls**. The data was expressed as mean ± SEM.

A total of 60 BA patients and 20 healthy controls were enrolled in this study. The characteristics of participants in both groups were demonstrated in Table 1. Mean age and gender ratio in controls and BA patients were not different whereas serum adiponectin levels in BA patients were markedly elevated compared with those in controls (15.5 ± 1.1 vs. 11.1 ± 1.1 μg/ml, *P *= 0.03) (Figure 1). Furthermore, BA patients had significantly higher hyaluronic acid than controls (50.3 ± 7.1 vs. 23.9 ± 1.5 ng/ml, *P *= 0.03). Additionally, liver stiffness scores in BA patients were dramatically higher than those in controls (30.1 ± 3.0 vs. 5.1 ± 0.5 kPa, *P *< 0.001).

**Table 1:** Demographic data, biochemical characteristics, and liver stiffness scores of controls and biliary atresia patients.

Variables	Controls (n = 20)	BA Patients (n = 60)	*P*-value
Age (years)	10.1 ± 0.7	9.6 ± 0.7	0.1
Gender (Female: Male)	10:10	32:28	0.5
BMI (kg/m^2^)	18.5 ± 0.5	18.0 ± 0.6	0.5
Albumin (g/dl)	-	4.3 ± 0.1	NA
Total bilirubin (mg/dl)	-	2.7 ± 0.5	NA
Direct bilirubin (mg/dl)	-	2.2 ± 0.5	NA
AST (IU/l)	-	128.1 ± 11.2	NA
ALT (IU/l)	-	109.5 ± 10.7	NA
ALP (IU/l)	-	431.0 ± 28.0	NA
Platelet count (10^3^/mm^3^)	-	162.7 ± 12.8	NA
APRI	-	3.0 ± 0.3	NA
Adiponectin (μg/ml)	11.1 ± 1.1	15.5 ± 1.1	0.03
Hyaluronic acid (ng/ml)	23.9 ± 1.5	50.3 ± 7.1	0.03
Liver stiffness (kPa)	5.1 ± 0.5	30.1 ± 3.0	<0.001

The data was expressed as mean ± SEM. BA, biliary atresia; BMI, body mass index; AST, aspartate aminotransferase; ALT, alanine aminotransferase; ALP, alkaline phosphatase; APRI, aspartate aminotransferase to platelets ratio index; NA, not applicable

### Comparisons between BA patients with and without persistent jaundice

**Figure 2:**
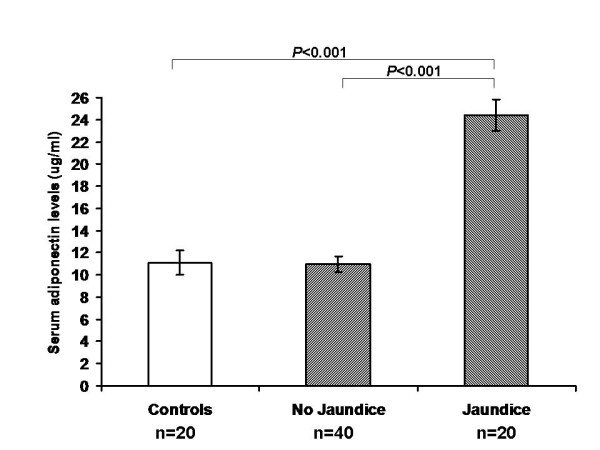
**Comparison of serum adiponectin levels in controls, biliary atresia patients without jaundice, and biliary atresia patients with jaundice**. The data was expressed as mean ± SEM.

We further categorized BA patients into jaundice (n = 20) and non-jaundice group (n = 40). As presented in Table 2, BA patients with jaundice had significantly higher serum bilirubin, AST, ALT, ALP, APRI and liver stiffness values compared to those without jaundice. Moreover, serum adiponectin levels in BA patients with persistent jaundice were greater than those in BA patients without jaundice (24.4 ± 1.4 vs. 11.0 ± 0.7 μg/ml, *P *< 0.001) (Figure 2). Similarly, BA patients with significant fibrosis (n = 44) possessed remarkably higher serum adiponectin than those with insignificant fibrosis (n = 16) (17.7 ± 1.2 vs. 9.4 ± 1.1 μg/ml, *P *< 0.001). We also found that BA patients with persistent jaundice had substantially higher levels of serum hyaluronic acid than those without jaundice (93.2 ± 16.7 vs. 28.9 ± 3.4 ng/ml, *P *< 0.001).

**Table 2:** Comparison between biliary atresia patients without and with jaundice.

Variables	BA Patients without jaundice (n = 40)	BA Patients with Jaundice (n = 20)	*P*-value
Age (years)	9.6 ± 0.9	9.5 ± 1.1	0.5
Gender (Female: Male)	20:20	12:8	0.5
BMI (kg/m^2^)	18.2 ± 0.6	17.1 ± 0.6	0.1
Albumin (g/dl)	4.5 ± 0.1	3.9 ± 0.1	<0.001
Total bilirubin (mg/dl)	0.7 ± 0.1	6.5 ± 1.2	<0.001
Direct bilirubin (mg/dl)	0.4 ± 0.1	5.9 ± 1.2	<0.001
AST (IU/l)	98.3 ± 11.6	187.7 ± 18.2	<0.001
ALT (IU/l)	94.1 ± 9.7	140.3 ± 24.5	0.04
ALP (IU/l)	369.8 ± 34.8	553.6 ± 33.9	<0.001
Platelet count (10^3^/mm^3^)	183.5 ± 15.5	121.2 ± 19.9	0.01
APRI	1.9 ± 0.4	5.0 ± 0.5	<0.001
Adiponectin (μg/ml)	11.0 ± 0.7	24.4 ± 1.4	<0.001
Hyaluronic acid (ng/ml)	28.9 ± 3.4	93.2 ± 16.7	<0.001
Liver stiffness (kPa)	18.5 ± 2.4	53.3 ± 4.3	<0.001

The data was expressed as mean ± SEM. BA, biliary atresia; BMI, body mass index; AST, aspartate aminotransferase; ALT, alanine aminotransferase; ALP, alkaline phosphatase; APRI, aspartate aminotransferase to platelets ratio index

**Figure 3:**
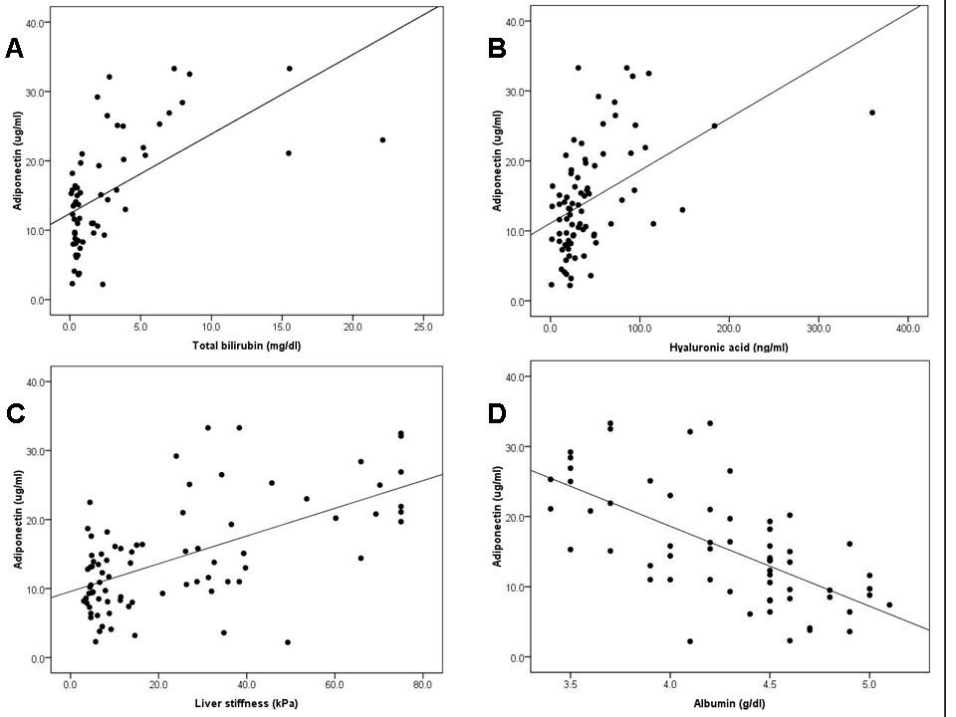
**Correlation analysis in biliary atresia patients**. Serum adiponectin is correlated with (A) total bilirubin (*r *= 0.58, *P *< 0.001), (B) hyaluronic acid (*r *= 0.46, *P *< 0.001), (C) liver stiffness (*r *= 0.60, *P *< 0.001) and (D) albumin (*r *= -0.65, *P *< 0.001) in patients with biliary atresia.

Further analysis demonstrated that serum adiponectin levels positively correlated with TB (*r *= 0.58, *P *< 0.001), serum hyaluronic acid (*r *= 0.46, *P *< 0.001), AST (*r *= 0.41, *P *= 0.001), ALP (*r *= 0.30, *P *= 0.02), and liver stiffness values (*r *= 0.60, *P *< 0.001). Conversely, serum levels of adiponectin inversely correlated with serum albumin (*r *= -0.65, *P *< 0.001). Correlations between serum adiponectin and total bilirubin, hyaluronic acid, liver stiffness, and serum albumin were shown in Figure 3.

## Discussion

Biliary atresia is an intractable liver disorder affecting infants and children. Despite early diagnosis and successful Kasai operation, the great majority of BA patients inevitably develop liver fibrosis, portal hypertension, and liver failure. Therefore, the investigation of fibrogenic progression in BA is undoubtedly important. Liver biopsy is considered a gold standard in diagnosing liver fibrosis and determining its severity. However, it is a painful and invasive procedure with infrequent but possible life-threatening complications [24]. Furthermore, there have been questions concerning the accuracy of liver biopsy, which is adversely affected sampling errors, intra- and inter-observer variability. These problems may result in false staging of liver fibrosis [25,22]. In an attempt to develop noninvasive methods for the assessment of liver fibrosis, transient elastography or FibroScan has emerged as the most promising tool in BA patients [26].

Transient elastography or FibroScan (Echosens, Paris, France) is a novel, rapid, and non-invasive technique for measuring the degree of liver fibrosis, and it can be performed in the out-patient setting. The transducer probe creates mild amplitude and low frequency (50 Hz) vibration, which induces an elastic shear wave in the tissues underneath. A pulse-echo ultrasound is used to follow the propagation of the elastic shear wave and to measure its velocity, which is in direct proportion to tissue stiffness [25,22]. According to the equation E = ρV^2 ^(E = Elastic modulus, V = Shear velocity, ρ = mass density), the stiffer the tissue is, the faster the wave can pass through it. FibroScan can measure liver stiffness in a volume of 100 times bigger than that obtained from liver biopsy and is, therefore, a better representative for the whole liver parenchyma [22].

Hepatic stellate cell activation followed by extracellular matrix production and accumulation is a major mechanism contributing to the development of liver fibrosis [6]. A number of cytokines are believed to play essential roles in this process and they become topics of research interest in an attempt to evaluate the use of serum cytokine as a biochemical marker of liver fibrosis. In the present study, we investigated the relationship of serum adiponectin with clinical outcomes and liver stiffness scores in postoperative BA patients.

Adiponectin - also known as complement-related protein 30 (Acrp30), adipose most abundant gene transcript (apM1) and adipoQ - is mostly synthesized by adipose tissue [27]. Various animal models and clinical researches showed that adiponectin mediated anti-obesity, anti-atherosclerotic, and anti-inflammatory effects [28]. Direct effects on hepatocytes via a specific receptor (AdipoR2 receptor) and anti-inflammatory properties are partly mediated by its antagonism against TNF-α [29]. This raises the postulation on the potential hepatoprotective role of adiponectin against liver fibrosis and cirrhosis. However, the possible role of adiponectin in the pathogenesis of BA remains as yet unclear.

The present study showed that serum adiponectin levels were significantly elevated in BA patients compared with healthy controls. In addition, serum adiponectin levels were substantially higher in BA patients with persistent jaundice than those without jaundice. Subsequent analysis revealed that serum adiponectin was positively correlated with serum total bilirubin, suggesting that serum adiponectin was associated with jaundice status in BA patients. Furthermore, jaundice status in BA patients seems to be an indicator for intrahepatic biliary obstruction. Thus, these findings indicate that adiponectin may play a potential role in the pathogenesis of hepatocellular damage in BA.

To our knowledge, this study demonstrates for the first time increased serum adiponectin levels in BA patients. We also found that serum adiponectin positively correlated with AST, ALP, hyaluronic acid, and liver stiffness, but negatively correlated with serum albumin. These results support that serum adiponectin is associated with clinical outcomes (jaundice status, hepatic dysfunction, and liver fibrosis) in BA patients post Kasai operation. Elevated serum adiponectin has been documented in various liver diseases, including acute hepatitis, chronic hepatitis, liver cirrhosis, hepatocellular carcinoma, and primary biliary cirrhosis [11,30-32]. In agreement with our findings, Tacke and colleagues demonstrated that adiponectin was increased and associated with inflammation and hepatic damage in chronic liver disease [11]. Elevated adiponectin concentrations following bile duct ligation in mice and in human bile from cholestatic patients suggest that biliary secretion is involved in adiponectin clearance. High adiponectin levels in patients with liver cirrhosis correlated positively with the severity of cirrhosis and negatively with hepatic protein synthesis, indicating that adiponectin might be used as a marker for liver cell injury [11,16]. These findings suggest that elevated circulating adiponectin is related to hepatic damage and hence reflects liver dysfunction. Accordingly, our results also revealed that serum adiponectin was positively associated with degree of liver stiffness determined using FibroScan. Liver stiffness values are well correlated with advanced stages of hepatic fibrosis and cirrhosis in children [33]. Wolf and coworkers showed that mice received adiponectin before concanavalin A treatment developed less hepatic damage [12]. Adiponectin expression was upregulated in concanavalin A-induced liver failure. Therefore, adiponectin could play a possible role in the regulation of hepatic inflammation. Future studies on HMW form of adiponectin may help identify more pieces of the inflammatory jigsaw of BA; nevertheless, the challenge remains to piece them together to originate a rational solid hypothesis pertaining to their exact role.

Several possible mechanisms may be responsible for the significant elevation of adiponectin in BA patients, particularly in those with a poor outcome. Increased serum adiponectin could be attributable to imbalance between adiponectin production and adiponectin clearance. In advance BA stages, reduced biliary clearance of adiponectin may plausibly contribute to elevated serum adiponectin levels. Moreover, extrahepatic organs can produce and secrete adiponectin in circulation. The higher adiponectin levels might be regarded as indicating hepatic injury and cholestasis in BA patients. Further clinical studies could render more valuable information on the pathophysiological roles of adiponectin in BA.

It should be noted, however, that there are some limitations in this study. Firstly, the sample size of participants was not large enough to arrive at definite conclusions. Secondly, we investigated only those subjects who attended King Chulalongkorn Memorial Hospital, a tertiary care center, for assessment or treatment of BA. As this investigation was designed as a cross-sectional study, therefore we could not determine the causal relationship between adiponectin and liver fibrosis. Other limitation would be the use of FibroScan instead of liver biopsy to evaluate the stage of liver fibrosis, which was not the definitive diagnosis. Furthermore, the sensitivity for significant fibrosis of 67% is not high enough to use Castera score as a screening tool of liver fibrosis in BA patients [22]. Prospective studies with a longitudinal design and hepatic expression of adiponectin would provide useful information on the role of adiponectin in hepatic fibrogenesis.

## Conclusion

This is the first study to demonstrate the elevation of serum adiponectin, hyaluronic acid, and liver stiffness values in BA patients. Serum adiponectin were correlated well with clinical parameters and the degree of liver fibrosis determined by FibroScan. Accordingly, serum adiponectin, hyaluronic acid, and transient elastrography could be used as non-invasive biomarkers reflecting the severity and progression of disease in BA patients post Kasai operation. Further studies will be needed to determine the precise role of adiponectin in the process of liver fibrogenesis.

## Abbreviations

ALT: alanine aminotransferase; ALP: alkaline phosphatase; APRI: aspartate aminotransferase to platelets ratio index; AST: aspatate aminotransferase; BA: biliary atresia; DB: direct bilirubin; HSC: hepatic stellate cells; TNF-α: tumor necrosis factor-α; TB: total bilirubin; kPa: kilopascals; HMW: high-molecular-weight.

## Competing interests

The authors declare that they have no competing interests.

## Authors' contributions

SH and MC have conceived the study, analyzed the data, and have written the manuscript. AT, KP, and WU performed laboratory analysis. VC, PV, and YP were involved in the diagnosis and recruitment of cases. YP was responsible for the design of the study. All authors read and approved the final manuscript.

## Pre-publication history

The pre-publication history for this paper can be accessed here:

http://www.biomedcentral.com/1471-230X/11/16/prepub
